# Construction and validation of cognitive frailty risk prediction model for elderly patients with multimorbidity in Chinese community based on non-traditional factors

**DOI:** 10.1186/s12888-023-04736-6

**Published:** 2023-04-18

**Authors:** Shuzhi Peng, Juan Zhou, Shuzhen Xiong, Xingyue Liu, Mengyun Pei, Ying Wang, Xiaodong Wang, Peng Zhang

**Affiliations:** 1 Graduate School, Shanghai University of Traditional Chinese Medicine, Shanghai, China; 2 Graduate School, Shanghai University of Medicine and Health Sciences, Shanghai, China; 3 Nursing Department, Funing People’s Hospital, Jiangsu, China; 4 ICU, Funing People’s Hospital, Jiangsu, China; 5 Department of Nephrology, Shuguang Hospital Affiliated, Shanghai University of Traditional Chinese Medicine, Shanghai, China; 6 School of Management, Hainan Medical University, Haikou, China

**Keywords:** Cognitive frailty, Non-traditional factors, Prediction model, Multimorbidity

## Abstract

**Background and objectives:**

Early identification of risk factors and timely intervention can reduce the occurrence of cognitive frailty in elderly patients with multimorbidity and improve their quality of life. To explore the risk factors, a risk prediction model is established to provide a reference for early screening and intervention of cognitive frailty in elderly patients with multimorbidity.

**Methods:**

Nine communities were selected based on multi-stage stratified random sampling from May–June 2022. A self-designed questionnaire and three cognitive frailty rating tools [Frailty Phenotype (FP), Montreal Cognitive Assessment (MoCA), and Clinical Qualitative Rating (CDR)] were used to collect data for elderly patients with multimorbidity in the community. The nomogram prediction model for the risk of cognitive frailty was established using Stata15.0.

**Results:**

A total of 1200 questionnaires were distributed in this survey, and 1182 valid questionnaires were collected, 26 non-traditional risk factors were included. According to the characteristics of community health services and patient access and the logistic regression results, 9 non-traditional risk factors were screened out. Among them, age OR = 4.499 (95%CI:3.26–6.208), marital status OR = 3.709 (95%CI:2.748–5.005), living alone OR = 4.008 (95%CI:2.873–5.005), and sleep quality OR = 3.71(95%CI:2.730–5.042). The AUC values for the modeling and validation sets in the model were 0. 9908 and 0.9897. Hosmer and Lemeshow test values for the modeling set were χ2 = 3.857, *p* = 0.870 and for the validation set were χ2 = 2.875, *p* = 0.942.

**Conclusion:**

The prediction model could help the community health service personnel and elderly patients with multimorbidity and their families in making early judgments and interventions on the risk of cognitive frailty.

## Introduction

The body’s metabolism slows down, brain cells progress to atrophy, and cognitive frailty is inevitable with advancing age. However, with the aging of the population and the prolonged life expectancy, Multimorbidity among the elderly is also common. Multimorbidity refers to a patient with at least two chronic diseases [1]. At the end of 2018, the ≥ 60-year-old population of China was 249.49 million, accounting for 17.9% of the total national population [2], and the proportion of the elderly with two or more chronic diseases was about 76.5%. Multimorbidity seriously threatens physical and mental health and reduces the quality of life of elderly patients. Frailty is a complex elderly syndrome characterized by age-related physiological stores and the frailty in the function of multiple systems, which lead to mild stress events and further negative health consequences [3]. It has been indicated that the incidence of combined frailty in the elderly > 65-years-old is about 10% and that in the elderly > 85-years-old is about 25–50% [4]. As one of the subtypes of asthenia, cognitive frailty is considered a clinical state wherein physical frailty and cognitive impairment coexist.

Prediction model provides individuals with the risk or probability of a specific event by combining multiple prediction factors and assigning each prediction factor the corresponding weight [5]. The nomogram model can be constructed easily and can predict the individual risk of cognitive frailty in elderly patients with multimorbidity. Previous prediction models for cognitive frailty mainly targeted traditional risk factors, such as total cholesterol, low-density lipoprotein, cholesterol, high-density lipoprotein cholesterol, triglycerides, hemoglobin, and albumin instead of the traditional risk, which is often neglected [6]. Non-traditional risk factors are common items in community diagnosis and treatment services, which can be obtained through consultation without increasing the cost and are applicable and accessible. Moreover, elderly patients have high control over these non-traditional risk factors [7]. A simple and feasible prediction model will also make the community health workers aware of the disease progression of multimorbidity with time and understand the factors underlying cognitive frailty. According to the prediction model, community health workers can provide preventive interventions for elderly patients with multimorbidity in the community, which are consistent with the characteristics of each patient before they develop the symptoms of cognitive frailty.

The incidence of cognitive frailty in elderly patients with multimorbidity is much higher than that in the general elderly [8, 9]. Cognitive frailty was first used in a 2001 study on the clock-mapping test [10]. Panza et al. [11] first proposed the theory of cognitive frailty in 2006. In 2013, the International Association of Gerontology and Geriatrics (IAGG) defined cognitive frailty as a heterogeneous clinical syndrome in the elderly characterized by the simultaneous presence of physical frailty and cognitive impairment but no Alzheimer’s disease or other types of dementia [12]. In 2015, Ruan et al. [13] divided the phenomenon into two subtypes: reversible and potential. The main difference between the two subtypes of frailty was in the manifestations of their cognitive dysfunction. Interestingly, the cognitive dysfunction of potential reversible cognitive frailty manifests as mild cognitive dysfunction, which is measured as CDR = 0.5 [14]. An interaction between asthenia and cognitive dysfunction is noted, and the development and changes of either side affect the other side, which together form a vicious circle of mutual influence. Compared with assessing weakness or cognitive dysfunction alone, assessing cognitive weakness can predicts the risk of adverse health outcomes in older adults.

Cognitive frailty is a non-specific syndrome in the elderly, with potential reversibility. However, if it is difficult to completely reverse the phenomenon, thereby necessitating the development of a convenient and rapid early warning tool. Xiao et al. [15] pointed out that vitamin D deficiency or insufficiency is positively correlated with cognitive frailty, while Chye et al. [16] showed that lack of vitamins C and E could easily lead to cognitive frailty in the elderly. However, these risk factors need to be detected by the instrument. In the studies on the cognitive frailty of elderly patients in China, many epidemiological surveys only target potential cognitive impairment, with a detection rate of 1.0%–50.1%. The studies on the elderly have large heterogeneity due to different regions and places [17]. Although a few studies have assessed the non-traditional risk factors of cognitive frailty in elderly patients with multimorbidity, those that can be identified early and easily intervened are yet unclear. Therefore, based on the community-accessible resources, this study explored the non-traditional risk factors of cognitive frailty in elderly patients with multimorbidity and constructed the risk prediction model based on a nomogram. This model provided the relevant basis for early recognition and delay of cognitive frailty in elderly patients with multimorbidity in the community, thereby improving their quality of life and reducing the family and social burden.

## Materials and methods

### Participants

Inclusion criteria: ① Age ≥ 60-years-old; ② Suffering from multimorbidity (two or more coexisting conditions in an individual); ③ Visits to community health service centers ≥ 2 times in the past 1 year. Exclusion criteria: ① Severe cognitive and mental disorders; ② Nervous system disease (such as cardiac, hepatic, and renal decompensation); ③severe visual and hearing impairment, dementia, and denial of participation.

### Instruments and measurements

Self-designed questionnaire included parameters such as gender, age, education level, marital status, whether living alone, physical exercise (square dancing, hiking, running, yoga, swimming, ball games, farm work, excluding housework, ≥ 2 times a week), intellectual activities (reading books and newspapers, writing, calligraphy, photography, painting, playing musical instruments, handicrafts, speculation, playing cards or mahjong, playing chess, surfing the Internet, on the University for the elderly ≥ 2 times a week), social activities(nearly one month), fall history(nearly one month), and the quality of sleep nearly one month (Poor sleep quality include difficulty in falling asleep, easy to wake up, difficulty in falling asleep after waking up, and fatigue in the morning were considered, Good sleep quality include fast falling asleep, deep sleep, awakening ≤ 2 times, easy to fall asleep again, and a clear and comfortable mind when waking up in the morning).

Frailty Phenotype (FP): It is used to assess whether the target population shows asthenia with respect to five aspects, such as decreased walking speed, decreased body weight, fatigue, decreased grip strength, and inability to walk forward. If one aspect is observed, it is scored as one point; if not, it is scored as 0 points. A total score of 0 points means no asthenia, 1–2 points indicate pre-asthenia, and 3–5 points mean asthenia. Cronbach’α and KMO value respectively are 0.897 and 0.890, and *P* value less than 0.05.

Montreal Cognitive Assessment (MOCA): It consists of eight parts and is used for cognitive screening of the target population. The total score is 30 points, with ≥ 26 points as normal and < 26 points indicating cognitive impairment. Cronbach’α and KMO value respectively are 0.839 and 0.895, and *P* value less than 0.05.

Clinical Dementia Rating (CDR): It is used to assess the degree of dementia in the target population. It consists of six items, based on memory and an additional five items for the five-level assessment of the target population. 0 means healthy, 0.5 means suspicious dementia, 1 means mild dementia, 2 means moderate dementia, and 3 means severe dementia. Cronbach’α and KMO value respectively are 0.890 and 0.898, and *P* value less than 0.05.

According to the International Academy of Nutrition and Aging (IANA) and the International Association of Gerontology and Geriatrics (IAGG) in 2013 [18]. Cognitive frailty was defined that the physical frailty (FP ≥ 3 points) and cognitive dysfunction (CDR = 0.5 and MoCA < 26 points) existed simultaneously, and Alzheimer’s disease and other types of dementia were excluded.

### Data collection

Using the multi-stage stratified random sampling method, three cities were randomly selected from Jiangsu Province in China, three counties were randomly selected from the three cities, and three communities were randomly selected from each county as survey sites. The survey was conducted during May 1 to June 1 2022. Two nursing researchers were trained by neurologists and passed the examination, and were familiar with assessment methods such as MoCA, CDR, and FP. The objectives and methods of this study were introduced to the elderly or their families in the community. After obtaining informed consent, face-to-face interview was conducted using a unified guiding language. During the investigation, the questions raised by the elderly were interpreted patiently and without suggestion. All items are filled in on-site, and the wrong or missing items were fed back and corrected in time. As the model usually has more parameters than actually contained variables, the events per candidate predictor parameter (EPP) principle was applied in this study [19, 20]. A total of 1200 questionnaires were distributed, and 1182 valid questionnaires were collected, with an effective recovery rate of 98.5%.

### Data analysis

Data analysis was conducted using SPSS 21.0, and the risk prediction model was constructed using Stata 15.0 was used to screen the variables and establish the model. The optimal critical value of the prediction model was calculated based on Jordan index, and the nomogram was drawn. The Bootstrap method was strengthened and the samples were re-sampled for 1000 times for internal verification. Participants were randomly divided into two groups at the ratio of 7:3, i.e., the ratio of 7 was in the modeling set and the ratio of 3 was in the verification set. The Hosmer–Lemeshow test (HL test) was used to evaluate the model fit and a P value greater than 0.05 indicated that there was no significant difference between the predicted and actual values by the HL test. The AUC value (> 0.7 indicated that the model had good resolution) was used to evaluate the prediction ability of the model, and the calibration curve were used to evaluate model calibration; inspection level α = 0.05.

## Results

### Descriptive analyses (Table 1)

**Table 1:** Demographic information

Variables	n (%)
Gender	
Female	700(59.22)
Male	482(40.78)
Age in years	
60 ~ 70	514(43.49)
> 70	668(56.51)
Education	
University and above	851(72.00)
High school and below	331(28.00)
Marital status	
Married	632(53.47)
Unmarried/divorced/widowed	550(46.53)

The cohort comprised 482/1182 (40.78%) males, and the age range was 60–97 (73.35 ± 7.99)-years-old. The level of education was divided into high school and above; 694/1182 (58.71%) lived alone; 550/1182 (46.53%) were unmarried/divorced/widowed. 461/1182 (39.0%) patients had no exercise habits, and 531/1182 (44.9%) had a history of falling. 406/1182 (34.3%) patients experienced cognitive frailty, including 293 (34.9%) cases in the modeling set and 113 (33.3%) cases in the verification set; no significant difference was observed in the detection rate (χ2 = 0.365, *p* = 0.546).

### Results of logistic regression analysis

**Fig. 1:**
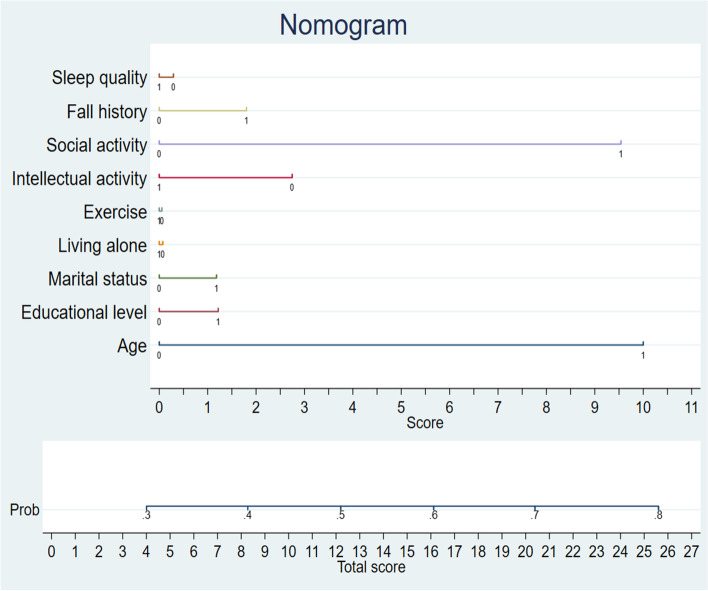
Nomogram of risk for cognitive frailty

In the modeling set, 26 non-traditional risk factors were subjected to univariate analysis. The variables with statistically significant differences shown in Table 2. Predictive factors with statistically significant differences in multivariate analysis were included in the model and expressed in nomogram (Fig. 1). Independent variable assignments are shown in Table 3.

**Table 2:** Results of logistic regression analysis

Variables	Modeling set HR (95% CI)		Verification set HR (95% CI)	
	Univariate analysis	Multivariate analysis	Univariate analysis	Multivariate analysis
Sex	1.109 (0.831–1.479)		1.055 (0.663–1.68) *	
Age	4.499 (3.26–6.208) **	2.711 (1.207–6.091) *	3.947 (2.332–6.68) **	3.561 (0.865–14.653)
Education level	0.010 (0.006–0.017) **	0.009 (0.003–0.026) **	0.009 (0.004–0.023) **	0.003 (0–0.029) **
Marital status	3.709 (2.748–5.005) **	8.333 (3.63–19.127) **	7.086 (4.203–11.949) **	39.816 (6.293–251.934) **
Living alone	4.008 (2.873–5.589) **	2.376 (1.041–5.423) *	3.986 (2.416–6.577) **	4.622 (1.211–17.641) *
Exercise status	0.019 (0.012–0.03) **	0.010 (0.004–0.028) **	0.103 (0.061–0.173) **	0.025 (0.005–0.122) **
Intellectual activity	0.335 (0.25–0.45) **	0.243 (0.108–0.545) **	0.380 (0.239–0.604) **	
Social activity	0.131 (0.095–0.181) **	0.087 (0.036–0.208) **	0.086 (0.05–0.148) **	0.109 (0.027–0.45) **
Fall history	4.791 (3.526–6.511) **	6.128 (2.786–13.478) **	5.506 (3.362–9.019) **	19.159 (3.574–102.709) **
Sleep quality	3.710 (2.73–5.042) **	6.371 (2.789–14.554) **	2.744 (1.7–4.429) **	6.449 (1.453–28.628) *

^*^ *p* < 0.05, ** *p* < 0.01

**Table 3:** Independent variable assignment

Variable	Evaluation	Variable	Evaluation
Sex	Male = 0, female = 1	Exercise status	No = 0, yes = 1
Age	60–70 years = 0, > 70 years = 1	Intellectual activity	No = 0, yes = 1
Educational level	Up to high school = 0, High school and below = 1	Social activity	No = 0, yes = 1
Marital status	In marriage = 0, unmarried, divorced, widowed = 1	Fall history	No = 0, yes = 1
Living alone	No = 0, yes = 1	Sleep quality	Poor = 0,Good = 1,

### Discrimination and calibration verification

**Fig. 2:**
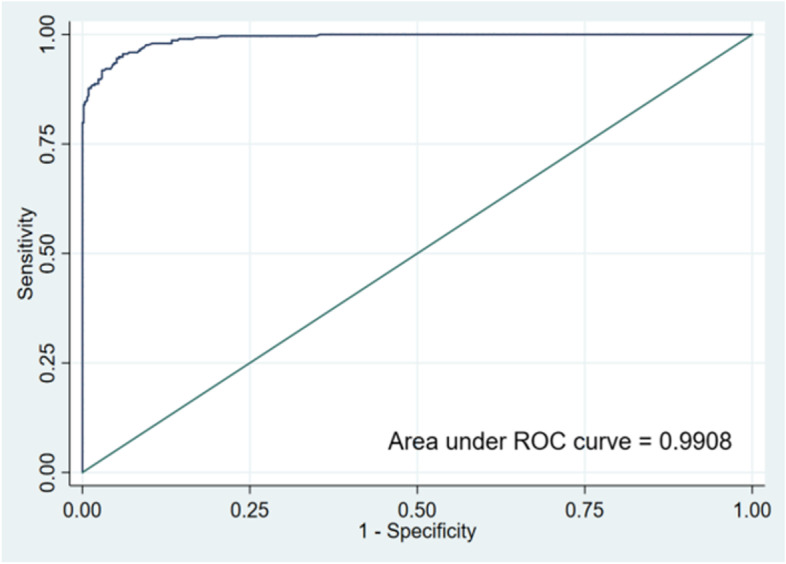
ROC curve for modeling set

**Fig. 3:**
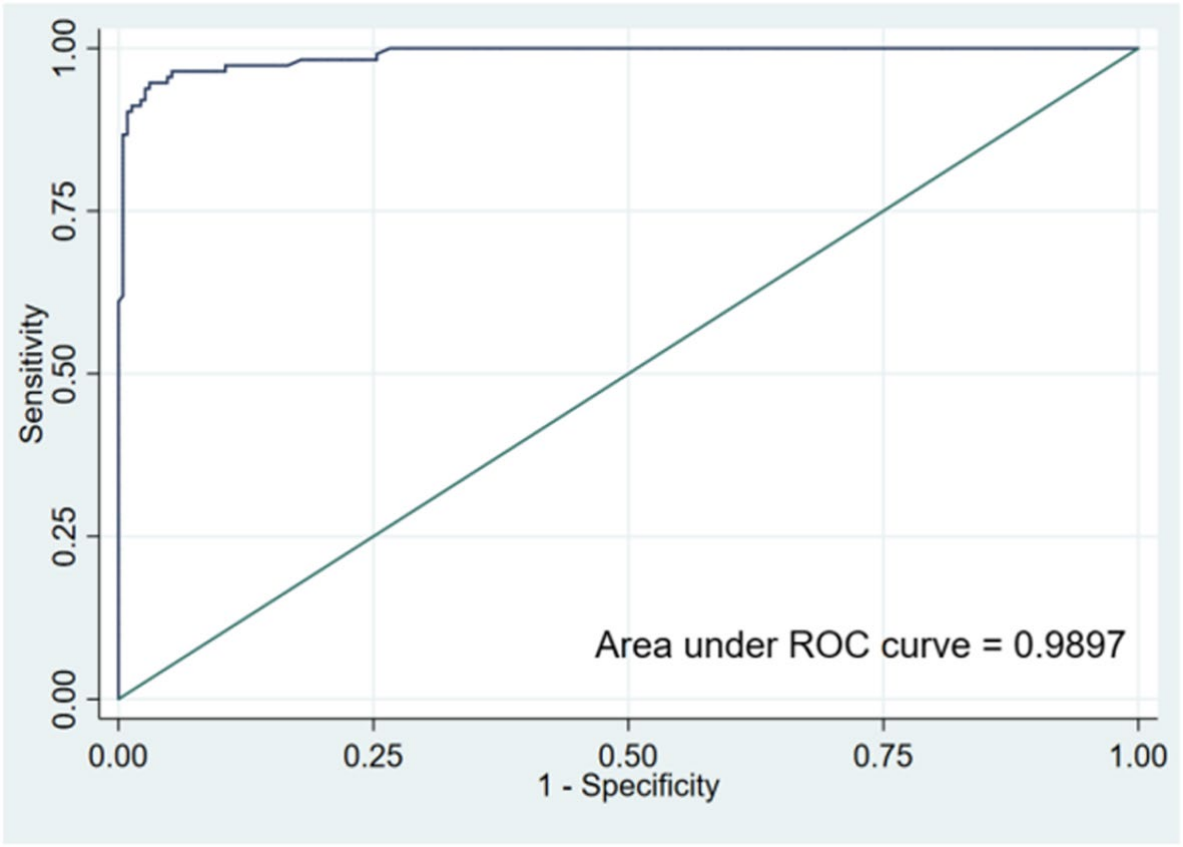
ROC curve for validation set

**Fig. 4:**
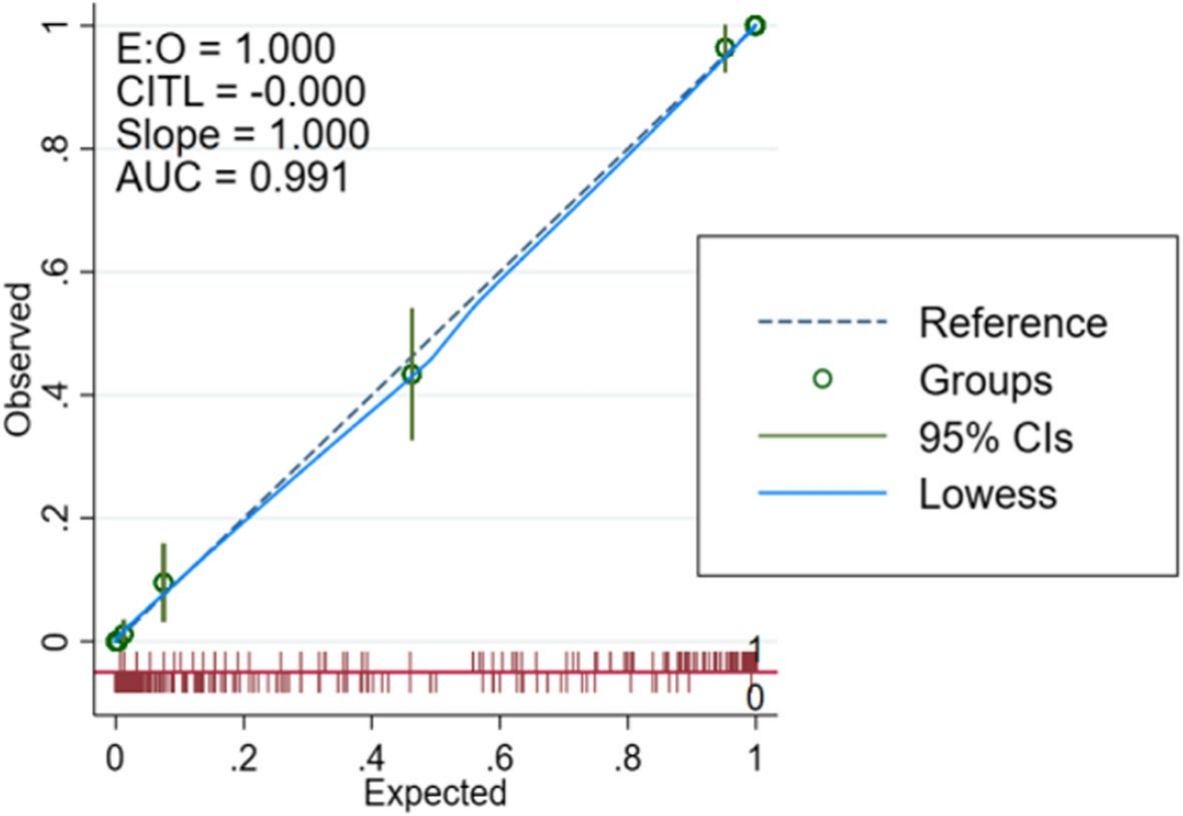
Modeling set calibration curve

**Fig. 5:**
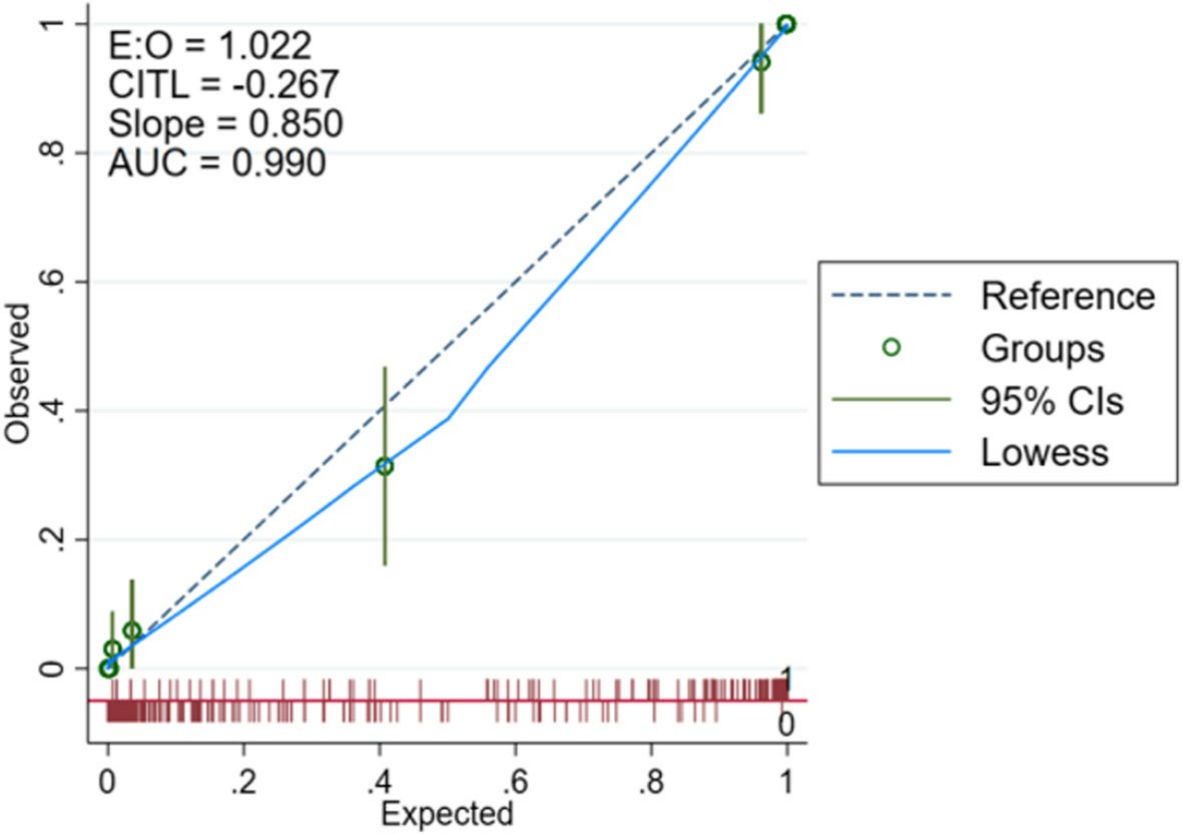
Verification set calibration curve

The area under the curve (AUC) values for the performance of the training and validation groups for the test nomogram were 0.9908 and 0.9897, respectively [training set: Hosmer and Lemeshow test (χ2 = 3.857, *p* = 0.870); validation set: Hosmer and Lemeshow test (χ2 = 2.875, *p* = 0.942)]. The receiver operating characteristic (ROC) curves are shown in Figs. 2 and 3. The nomogram calibration curves predicting the risk of cognitive frailty in elderly patients with multimorbidity in the community showed good consistency between the training and verification group (Figs. 4 and 5).

### Clinical application

**Fig. 6:**
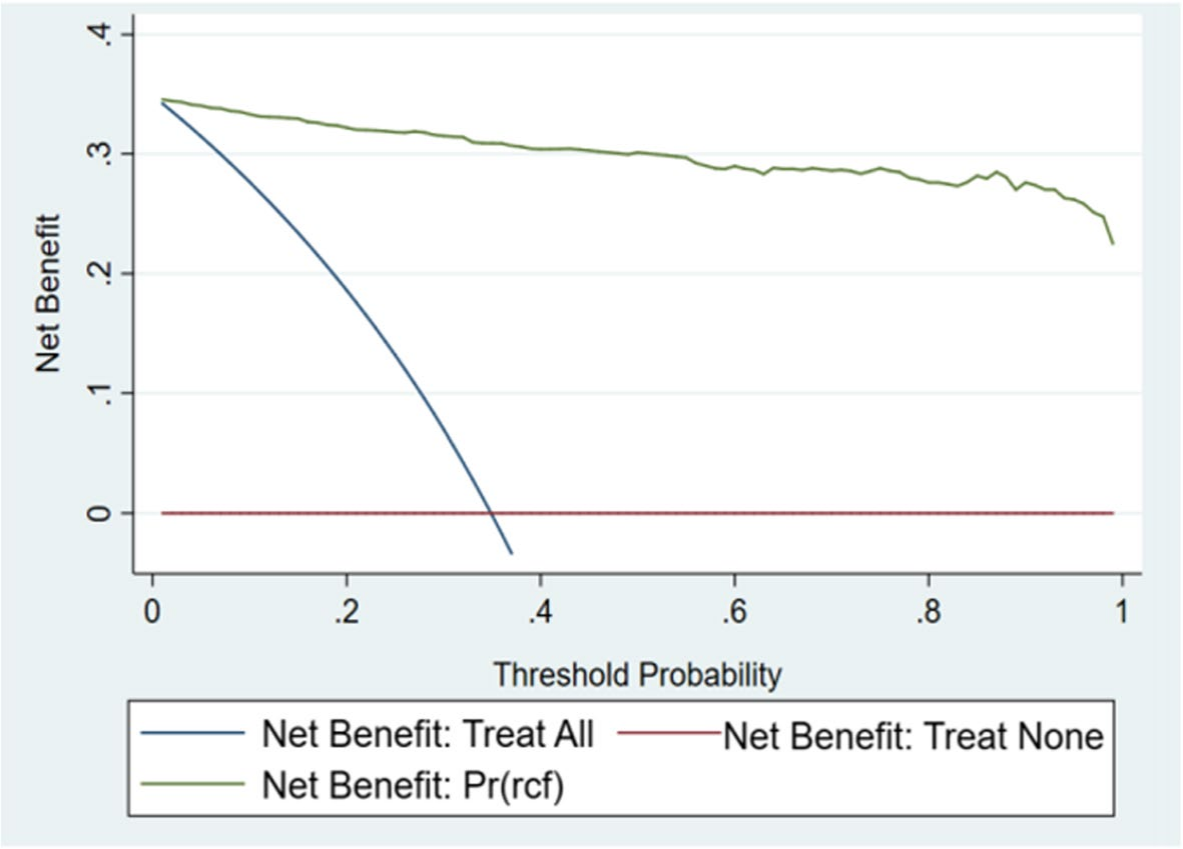
Decision curve of modeling set

**Fig. 7:**
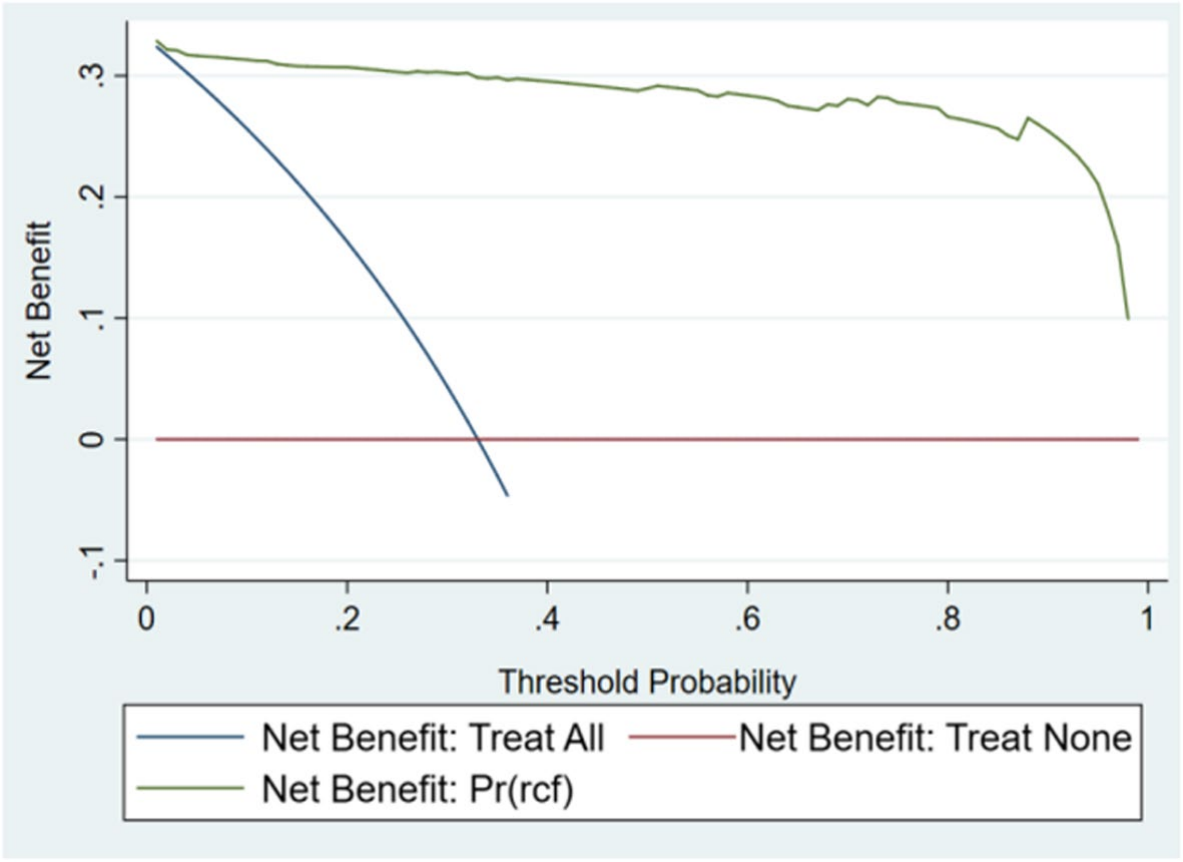
Decision curve of verification set

The analysis of decision curves of the nomogram of the clinical application in the modeling and verification groups is shown in Figs. 6 and 7.

## Discussion

The current results showed that the detection rate of cognitive frailty in elderly patients with multimorbidity in the community was 34.3%, which was similar to the screening results (35.0%) of Merchant et al. [21]. Statistics from the United States showed that two-thirds of the elderly suffer from multimorbidity. Moreover, 27% of patients with multimorbidity expend a lot of medical resources (67% of the total population). The problem of multimorbidity is becoming prominent [22]. The progression of cognitive frailty accelerates the disability, hospitalization, and mortality rates of elderly patients with multimorbidity [4, 23, 24]. Therefore, establishing a risk prediction model to identify early cognitive frailty in elderly patients with multimorbidity is essential for timely intervention. In our study, 9 indicators were screened out by logistic regression, and a visual nomogram prediction model was established. Together, these findings indicated that the model had sufficient judgment ability and clinical efficiency for predicting the risk of cognitive frailty.

The pathogenesis of cognitive frailty is complex. In previous research on forecasting models, traditional risk factors usually be obtained through professional instruments and equipment or diagnosis by a professional physician [25]. In our study, the non-traditional risk factors are common items in community diagnosis and treatment services, which could be obtained through inquiry without increasing the costs; this approach has good applicability and accessibility [26]. The results showed that age, education level, marital status, living alone, exercise, intellectual activity, social activity, fall history, and sleep quality are the independent risk factors for cognitive frailty in elderly patients with multimorbidity [27]. A certain degree of inevitable physiological brain aging will appear in the elderly with age, thus accelerating the occurrence and development of cognitive frailty [28]. As an independent risk factor affecting the physical and mental health of the elderly, living alone has a significant impact on their cognitive function. Related studies have shown that due to the reduced communication activities with their families, the elderly living alone receive less cognitive stimulation in the brain, and the connectivity of neural networks is not strong, thus resulting in weak cognitive function [29]. These results indicated that the incidence of cognitive frailty in participants without intellectual activity was higher than in those with intellectual activity. In our study, the incidence of cognitive frailty in participants with a history of falls was higher than that in patients without a history of falls, similar to the results reported by Kim et al. [29]. Some studies have shown that active physical exercise has a negative correlation with the occurrence of cognitive frailty in the elderly [30], i.e., the higher the frequency of physical exercise in the elderly, the lower the incidence of cognitive frailty, which is in agreement with our findings. This phenomenon could be attributed to physical exercise conducive to enhancing muscle strength and delaying osteoporosis. Importantly, it can reshape brain function and reduce the risk of brain atrophy [31]. The prevalence of sleep disorders in the elderly in China is 41.2% [32]. Another study [33] has shown that sleep disorders are a major risk factor for cognitive frailty. Insomnia is one of the common chronic diseases in elderly patients, and the coexistence of multiple prolonged diseases makes elderly patients suffer from both physical and psychological pressure for a long time. In our study, bad sleep quality had a high probability of cognitive frailty, indicating that cognitive frailty could be screened quickly by sleep quality [34]. Therefore, the community health service center staff and the families in participants should be involved in the active prevention and treatment of chronic diseases in the elderly. Community health service centers should deliver lectures on the prevention and treatment of cognitive frailty, and carry out regular screening to identify cognitive frailty within the scope of physical examination to reduce or delay the occurrence and development of the condition.

In addition, the nine predictive factors included in the model were common items in community diagnosis and treatment services, and the consultation was dominant. The data were easily obtained without increasing the economic cost. The elderly patients with multimorbidity had high compliance and good applicability and accessibility. The predictive risk value of the cognitive frailty for the elderly in the community was obtained. Distinguishing the high-risk and low-risk groups based on the optimal threshold screens for cognitive frailty in the early stage while avoiding wastage of medical resources.

### Implications for clinical practice

Presently, only a few studies are available on risk prediction models for non-traditional risk factors of cognitive frailty in elderly patients with multimorbidity in China. In this study, the nomogram model was constructed based on the characteristics of the Chinese population by incorporating non-traditional risk factors. These features rendered the model simple and feasible, such that it could predict the individual risk of cognitive frailty in elderly patients with multimorbidity and provide support for early recognition and intervention. In the secondary prevention of chronic diseases, the development of prediction models using interventional, low-cost, and easily accessible variables can achieve “early detection, early diagnosis, and early treatment” of cognitive frailty in community health centers. In the tertiary prevention of the disease, predictive models can be used to predict relapse and thus reduce mortality and disability [35]. A simple and feasible prediction model would also make the community health workers aware of the disease progression of senile patients with multimorbidity as the disease changes over time and find the factors that may cause the cognitive frailty [36, 37].

### Strengths and limitations

Although the construction effect of this prediction model is satisfactory, there are still some deficiencies. Firstly, this was a cross-sectional study, and the causal correlation between the investigated factors and cognitive frailty could not be determined. Secondly the samples of this study were only from Jiangsu Province, China, their nationwide or international application needs further external verification by a multi-center study. Although the non-traditional factors selected in this study are easy to obtain, do not increase the economic cost, and have good applicability and accessibility, but compared with the traditional factors, it is not so direct and accurate in disease risk prediction.

## Conclusions

The prediction model constructed could help the community health service personnel and elderly patients with multimorbidity and their families in making early judgments and interventions on the risk of cognitive frailty. It is necessary to focus on the elderly who have poor sleep quality and live alone, and appropriate exercise plan should be formulated according to the physical condition of the elderly patients.

## Data Availability

The datasets used and analyzed in this study are available from the corresponding author on reasonable request. The data are not publicly available due to their containing information that could compromise the privacy of research participants.

## Abbreviations

CF: Cognitive Frailty
FP: Frailty Phenotype
MoCA: Montreal Cognitive Assessment
CDR: Clinical Qualitative Rating
OR: Odds Ratio
AUC: Area Under ROC Curve
CI: Confidence Intervals
EPP: Events Per Candidate Predictor Parameter
